# Serum Vitamin D Is Associated with Antioxidant Potential in Peri-Parturient Cows

**DOI:** 10.3390/antiox10091420

**Published:** 2021-09-06

**Authors:** Jaimie M. Strickland, Lauren Wisnieski, Vengai Mavangira, Lorraine M. Sordillo

**Affiliations:** 1 Large Animal Clinal Sciences, College of Veterinary Medicine, Michigan State University, East Lansing, MI 48824, USA; strick51@msu.edu (J.M.S.); mavangir@msu.edu (V.M.); 2 Center for Animal and Human Health in Appalachia, College of Veterinary Medicine, Lincoln Memorial University, Harrogate, TN 37752, USA; lauren.wisnieski@lmunet.edu

**Keywords:** oxidative stress, vitamin D, dairy cows

## Abstract

Dairy cows experience increased oxidative stress during periods of transition such as at the cessation of lactation and around the periparturient period, thus increasing disease risk. Despite routine supplementation of transition cow diets with certain vitamins in an attempt to mitigate oxidative stress, there is no currently available data directly linking vitamin supplementation with antioxidant potential (AOP) in transition cows. The objective of this study was to determine the association between serum vitamins and biomarkers of oxidative stress in healthy cows. Blood samples were collected from 240 cows at dry off (DO), close up (CU), and 2–10 days post-calving (DIM2-10). Blood samples were analyzed for vitamins (A, D, E), β-carotene, reactive oxygen species (ROS), and AOP. Spearman correlations and mixed linear regression models were used to assess associations between vitamins and measures of oxidant status. Vitamin D concentrations were positively associated with AOP at the CU and DIM2-10. Based on the positive association with AOP, additional in-vitro studies were conducted that showed vitamin D mitigated barrier integrity loss in endothelial cells during oxidative stress. These results indicate for the first time that vitamin D may have a role in promoting antioxidant potential in transition dairy cows.

## 1. Introduction

Dairy cattle are particularly susceptible to oxidative stress during the peripartum period because of metabolic and endocrine challenges occurring during parturition, lactogenesis, and external stressors such as pen and dietary changes [1]. Oxidative stress is caused from an imbalance of reactive oxygen species (ROS) and antioxidant potential (AOP) [2]. ROS are oxygen-containing compounds that donate electrons to molecules in cells causing damage and dysfunction, whereas AOP encompasses a variety of enzymes and compounds that neutralize radicals and provide protection for cells [3]. Oxidative stress is the term that refers to the damaged proteins, DNA, or lipids when ROS overwhelm AOP’s neutralizing capabilities [4]. Oxidative stress is increased in dairy cattle during the periparturient period and may play a role in the pathogenesis of periparturient diseases [2,5,6]. Indeed, there is increasing focus on supplementing late gestation dairy cows with vitamins and minerals to prevent oxidative stress and thus decrease the risk for periparturient diseases [7].

Decades of research have demonstrated the benefits of feeding antioxidant supplements such as vitamin E and selenium to dairy cows [8,9]. Selenium and vitamin E in pre-partum diets reduced the incidence of retained placenta, improved fertility, and decreased cases of clinical mastitis [10,11,12]. Also, vitamin E and selenium improved immune responses through enhanced neutrophil function and increased antioxidant capacity [8,13]. More recently, research focused on the impact of vitamin E and selenium to reduce in-vitro models of oxidative stress in bovine aortic and mammary endothelial cells [14,15]. Collectively, the existing literature supports the widespread use of vitamin E and selenium supplementation in periparturient cows to decrease risk for disease [16]. Despite the body of literature on enhancing immune function and improving redox balance with supplemental vitamin E and selenium, dairy cows still experience oxidative stress in the periparturient period which further increases the risk for disease [17]. Indeed, approximately 45–75% of disease incidence occur during the first 30 d in milk when cows are at highest risk for oxidative stress [18,19]. Thus, alternative methods for reducing oxidative stress and disease risk in the periparturient period are needed.

In addition to vitamin E and selenium, supplemented or naturally occurring vitamins such as -A, -D, and β-carotene in the diets of dairy cows demonstrated the ability to optimize immunity and antioxidant defenses [20,21,22]. Indeed, human studies found that vitamins A, D, and β-carotene have critical roles in reduced oxidative stress and improved disease risk and progression [23]. However, during the time-period for increased risk for oxidative stress for dairy cattle in the periparturient period, vitamins A, D, and β-carotene reached their lowest serum concentrations and tended to remain low through the critical transition period [24,25]. Moreover, decreased serum vitamin concentrations in the periparturient period are related to increased risk for disease morbidity [26]. For example, decreased serum vitamin A concentrations in the periparturient period were associated with increased risk of mastitis, uterine diseases, and hyperketonuria [26,27]. Supplemental β-carotene reduced the incidence of retained placenta [28]. Decreased concentrations of serum vitamin D were associated with increased risk for uterine disease, but conversely presence of urine ketones was associated with elevated concentrations of vitamin D [29]. Although the pathology behind these findings were unknown, the authors suggested that increased serum vitamin D concentrations improved the formation of ketone bodies and subsequent hyperketonuria [29]. Indeed, increased dietary vitamin D supplementation tended to increase serum concentrations of beta-hydroxybutyrate [30]. Further, Wisnieski et al., 2020 suggested that the association between decreased serum vitamin D concentrations at 2–10 days post-parturition and uterine disease was bi-directional in that increased metabolism of vitamin D resulted from the immune response to the uterine diseases and that decreased serum vitamin D can decrease immune cell function [29]. However, the association between serum concentrations of vitamins A, D, and β-carotene with biomarkers of oxidative stress in the periparturient period remained unknown. Therefore, the objective of this research was to determine the associations between vitamins A, D, E, and β-carotene with biomarkers of oxidative stress and antioxidant potential. We hypothesized that serum concentrations of vitamins A, D, E, and β-carotene would be negatively associated with biomarkers of oxidative stress in periparturient cows.

## 2. Materials and Methods

### 2.1. Animals

A total of 353 cows from 5 commercial Michigan dairy herds were enrolled from September 2014 to August 2018. Cows were fed total mixed rations formulated to meet their nutritional requirements according to the National Research Council [16]. Exact dietary vitamin supplementation information for each farm can be found in Strickland et al., 2021 and Wisnieski et al., 2020 [27,29]. No injectable vitamin supplementation was given to any animal during the course of this study. Cows were enrolled in cohorts of up to 15 animals/cohort evenly divided between heifers < 25 mo old, second-parity cows, and third or greater parity cows. Cows and heifers were randomly selected at enrollment when cows and heifers were approximately 200 to 230 days pregnant, and cows had fewer than 380 days in lactation. Exclusion criteria for the current study included diagnosis of any disease from the time of enrollment to 30 days post-parturition. These diseases included metritis, retained placenta, mastitis, milk fever, lameness, displaced abomasum, ketosis, pneumonia, abortion, and death. Cows were monitored daily by trained farm staff using the disease definitions described in Strickland et al., 2021 [27]. Briefly, metritis was diagnosed by the presence of abnormal vaginal discharge, rectal temperature > 39.4 °C, and a flaccid uterus. A cow was diagnosed with a retained placenta if fetal membranes failed to be voided within 24 h of calving. Mastitis was defined as the presence of abnormal milk. Cows were diagnosed with milk fever when they presented with muscle tremors, subnormal body temperature, or muscle weakness that could result in the inability to rise. The lameness scoring system developed by Sprecher et al., 1997 was utilized to diagnose cows with an abnormal gait, corresponding to a score of 3 or greater out of 5 [31]. A displaced abomasum was identified by a ping heard with simultaneous auscultation with percussion between the 9th and 12th rib. A cow was diagnosed with ketosis when urine acetoacetate concentrations exceeded 1.5 mmol/L measured by Ketostix (Bayer AG, Leverkusen, Germany). Pneumonia was identified by altered breathing patterns and abnormal lung sounds upon auscultation. Abortion was defined by identifying a nonpregnant cow that had previously been diagnosed as pregnant [27]. This resulted in 240 healthy cows that were included in our investigation into the association of serum fat soluble vitamins and oxidative stress.

### 2.2. Sample Collection

Blood samples were collected from the coccygeal vein at dry off (DO) (−48 ± 12 d pre-calving), close-up (CU) (−17 ± 7 d pre-calving), and fresh (DIM2-10) (7 ± 3 d post-calving) in serum or plasma EDTA tubes (BD Vacutainer, Franklin Lakes, NJ, USA). Blood tubes were transported to the laboratory on ice but were then allowed to clot at room temperature for approximately 1 h. Serum was separated by centrifugation at 2000× *g* for 20 min at 20 °C. Aliquots of serum were placed into 1.5 mL microcentrifuge tubes and stored at −20 °C until analysis. To prevent ex-vivo lipid peroxidation in samples for oxylipid analysis, an antioxidant reducing agent was mixed with plasma samples both prior to freezing and post-thawing as previously described [32]. These samples were flash frozen in liquid nitrogen and stored at −80 °C until analysis.

### 2.3. Vitamin Analysis

All samples were analyzed within 1 to 6 months of collection to prevent vitamin degradation [33]. The concentrations for vitamin A, vitamin E, and β-carotene were analyzed at the Michigan State University Veterinary Diagnostic Laboratory (Lansing, MI, USA) by liquid chromatography mass spectrometry (LC/MS) utilizing a method by Arnaud et al. (1991) and were well described in Strickland et al. (2021) [27,34]. Briefly, five-point calibration curves were constructed using stock alpha-tocopherol (Sigma-Aldrich, St. Louis, MO, USA) solution (absorbance of 0.09 to 0.11 at 292 nm), retinol standard (Sigma-Aldrich, St. Louis, MO, USA) (absorbance of 0.085 to 0.095 at 325 nm), and beta-carotene standard (Sigma-Aldrich, St. Louis, MO, USA) (absorbance of 0.18 to 0.22 at 450 nm). Apocarotenal (Trans-β-APO-8”-carotenal, Fluka, St. Louis, MO, USA) was dissolved in methylene chloride to an absorbance of 0.10 to 0.15 at 450 nm to make the internal standard solution. Samples were analyzed by LC/MS using a Waters Acquity system and Waters Empower Pro Chromatography Manager software (Waters Corporation, Milford, MA, USA). Peak integration was by the ApexTrack method of Empower Pro with manual review. Serum vitamin D was analyzed by radioimmunoassay by Heartland Assays (Iowa State University Research Park, Ames, IA, USA) in the same manner as Holcombe et al. (2018) [25,35]. Briefly, the detection range was 2.5–100 ng/mL, the interassay and intraassay coefficients of variation were 13.3 and 6.5% respectively, and the assay was validated by serial dilution and tested for linearity [25].

### 2.4. AOP Analysis

The AOP was quantified in serum samples as described previously [36]. The monocation, 2,2′-azinobis-(3-ethylbenzothiazoline-6-sulfric acid) (ABTS) (SigmaAldrich, St. Louis, MO, USA) was oxidized by potassium persulfate to create a stable radical in a glass container that was sealed and covered with foil to protect it from light exposure [36]. The ABTS radical solution was used between 12 and 24 h of production and was then discarded after each use. The AOP of a sample was standardized to the reduction capacity of Trolox (6-hydroxy-2,5,7,8-tetramethylchroman-2-carboxylic acid or synthetic vitamin E analog) (SigmaAldrich, St. Louis, MO, USA) which was diluted to create a 6-point standard curve at concentrations of 25 µM, 12.5 µM, 6.25 µM, 3.125 µM, and 1.56 µM. The known reducing capacity of Trolox was used to determine the reducing capacity of each sample. The dilution of ABTS in phosphate buffered solution (PBS) was determined by analyzing serial dilutions (1:70, 1:75, 1:80, 1:85, 1:90, 1:95) in triplicate in colorimetric plate reader (Tecan, Mannedorf, Switzerland) at 730 nM for concentration of 0.7 ± 0.02. Samples were diluted 1:20 in PBS prior to being plated in triplicate with the standards. Once ABTS was added to wells, plates were covered with aluminum foil and incubated for 6 min and read at 730 nM. For the present study, the interassay and intraassay coefficient of variance were 4.2% and 0.92%, respectively.

### 2.5. ROS Analysis

The pro-oxidant concentration of serum samples, or ROS, was determined by the d-ROMS assay (Diacron International, Grosseto, Italy). The d-ROMS assay is an indirect measurement of hydrogen peroxide by addition of N,N-diethylparaphenylinediamine. This substrate forms a stable cation with hydrogen peroxide derivatives [37]. The results are expressed as “Carratelli units” (CarrU) with 1 CarrU having the equivalent oxidizing capacity as 0.08 mg/dL of hydrogen peroxide.

### 2.6. LC-MS/MS Analysis

For this analysis, 7 cows were selected from the group of 220 healthy cows. Because ex-vivo alterations in oxylipids occurred over time in storage and this study was carried out over the course of 3 years, only cows sampled within the final 6 months were used [38]. Of the 25 cows in this cohort, 9 were excluded for having 1 or more disease, and of the remaining 16 cows, only 7 had all 3-sample time points available for analysis. Targeted oxylipids were analyzed with liquid chromatography tandem mass spectrometry (LC-MS/MS). Similarly, the mixture of deuterated internal standards including 5(S)-hydroxyeicosatetraenoic-d8, 15(S)-hydroxyeicosatetrsenoic-d8 acid, 8(9)-epoxyeicosatrienoic-d11 acid, prostaglandin E2-d9, arachidonic acid-d8, 8(9)-epoxyeicosatrienoic-d11 acid, 2-arachidonoyl glycerol-d8, and arachidonoyl ethanolamide-d8 was prepared in the same manner as Mavangira et al., 2015 with final concentrations of 0.25, 0.25, 0.5, 0.5, 50, 2, and 0.25 µM and added to every sample [32]. This mixture was then used to create a 6-point standard curve ranging from 0.001 to 500 µM. First, frozen plasma samples were thawed on ice and then 2 mL of plasma from each sample was combined with 5 mL, methanol, 2 µL of formic acid, and 15 µL of internal standard mixture. Samples were vortexed for 2 min, allowed to incubate for 15 min at room temperature, and then were centrifuged for 20 min at 4 °C and 4816× *g*. The supernatant was diluted with 190 µL of 50:50 HPLC-grade water and formic acid. Prior to extraction, solid-phase extraction columns (Waters, Milford, MA, USA) were conditioned with 6 mL of methanol and then 6 mL of high-performance liquid chromatography (HPLC) water. Samples were added to columns and washed with 20% methanol and then 4 min of vacuum was used to fully dry the columns. A 50:50 mixture of methanol:acetonitrile was used to elute the samples which were then dried under vacuum using a Savant SpeedVac at 45 °C for at least 3 h run-time or until dried (ThermoQuest, Holbrook, NY, USA). The dried residues were reconstituted using 150 µL of 2:1 methanol:water and transferred to chromatography vials with inserts and stored at −20 °C until analysis. The quantification of analytes was performed on a Waters Acquity HPLC with a Waters Xevo TQ-S triple quadrupole mass spectrometer (Waters, Milford, MA, USA). Analyte separation was accomplished using an Ascentis Express C18 HPLC column, 10 cm × 2.1 mm, 2.7 µm (Supelco, Bellefonte, PA, USA). Samples were held at 50 °C, mobile phase A was water with 0.1% formic acid, mobile phase B was acetonitrile and a flow rate of 0.3 mL/min was maintained. Ratios of mobile phase A:B were as follows: 99:1 for time 0 to 0.5 min, 60:40 at 2.0 min, 20:80 at 8.0 min, 1:99 at 9 min until 13.0 min, and then 99:1 from 13.1 min until 15 min at the end of liquid chromatography separation for each sample. Oxylipids were detected in negative ion mode using electrospray ionization. Each oxylipid was analyzed with QuanOptimize software (4.0, Waters, Milford, MA, USA).

### 2.7. Cell Culture and Treatments

Cell culture experiments using bovine aortic endothelial cells (BAEC) were perfomed using established protocols for inducing oxidative stress in-vitro [39]. The BAEC were chosen because of their central role in immune responses and their location at the vascular and tissue interface where they are targets of prooxidant damage during oxidative stress. The BAEC were harvested previously from sections of aortas collected immediately after slaughter using methods previously described by our group [40]. Pro-oxidant challenge of BAECs was induced using 2,2′-azobis-2-methyl-propanimidamide, dihydrochloride (AAPH, Cayman Chemical, Ann Arbor, MI, USA). Treatments with calcitriol (1α,25-Dihydroxycholecalciferol/1α,25-DihydroxyvitaminD3/25-(OH)_2_-D_3_, Biovision, Milpitas, CA, USA) were performed to assess the effect of vitamin D on the AAPH-induced pro-oxidant challenge. The working doses of AAPH and vitamin D were determined by assessing cellular viability based on quantifying ATP production using the Cell Titer-Glo assay (Promega Corp., Madison, WI, USA) as previously described [39].

### 2.8. mRNA Quantification in BAEC

The quantification of gene expression of target genes was performed as previously described by our group [41]. The RNA from BAECs was extracted using the (Promega Maxwell RSC instrument, Madison, WI, USA) following the manufacturer’s protocol. Quantitative real-time PCR (qRT-PCR) was performed using predesigned and custom-designed TaqMan primers from Applied Biosystems (St. Louis, MO, USA) quantitative real-time (qRT-PCR) was performed. The qRT-PCR was performed in triplicate for each sample using reaction mixtures containing actin beta (ACTB); Glucuronidase beta (GUSB), and ribosomal protein S9 (RPS9) as endogenous controls. The cytochrome P (CYP) 24A1, nuclear erythroid factor 2 like 2 (NFE2L2), and vitamin D receptor (VDR) were the target genes (Table 1). The thermal cycling conditions for fast 2-step PCR were used. Stage 1, enzyme activation at 95 °C for 20 s; stage 2, 95 °C for 3 s; stage 3, 60 °C for 30 s; with 40 replications through stages 2 and 3. The abundance of target genes was normalized to endogenous control genes and calculated using the ∆Ct method for statistical analyses. The 2^−∆∆Ct^ method for relative expression was used to display the data [41].

### 2.9. Endothelial Cell-Substrate Impedance Sensing (ECIS)

Barrier integrity of BAEC was performed using a protocol previously described by our group [42] with minor modifications. Briefly, BAEC were cultured in 96-well arrays with a 10+ electrode system until approximately 80% confluency in the 5% serum-containing media. Cells were preincubated with 10 nM of vitamin D for 12 hours before adding AAPH at 3 mM. Resistance to electrical passage across the confluent monolayer was monitored using the electric cell-substrate impedance sensing system (ECIS, Applied Biophysics, Inc., Troy, NY, USA). Resistance measurements immediately before treatment additions were used to normalize all subsequent values.

### 2.10. Data Analyses

Cows that had one or more of the following health events during the study period were excluded: metritis, mastitis, ketosis, lameness, displaced abomasum, retained placenta, pneumonia, milk fever, abortion, death, and/or death of calf. Stata version 14.2 (College Station, TX, USA) was used for all analyses.

Mixed linear regression models for vitamins and AOP. Mixed linear regression models were built to assess factors associated with AOP. Three models were built: one for DO, one for CU, and one for DIM2-10. Candidate variables for the models included the four vitamin biomarkers (β-carotene, vitamin A, vitamin D, and vitamin E), parity, year (to adjust for differences between the two machines used), and all possible two-way interactions. A backwards selection was performed to select the variables in the final model (alpha = 0.05). Multicollinearity was carefully monitored throughout the model building process by assessing standard errors for inflation and by calculating the variance inflation factors (VIFs). Normality of residuals was visually checked using histograms and Quartile-Quartile (Q-Q) plots. If normality of residuals was violated, then the outcome variable was transformed (e.g., log base 10 function). A random intercept for farm and/or cohort was included unless the estimates were very small and did not affect the other model coefficients when they were excluded.

Spearman correlation statistics for association of vitamins with oxylipids. 20-HETE, and ROS. Spearman correlation statistics were calculated to assess the association of each vitamin (β-carotene, vitamin A, vitamin D, and vitamin E) with multiple oxylipids including 5-isoprostaglandin F2α VI (5-iPF2alphaVI), 8,12-isoprostane, 8-isoprostane prostaglandin A2 (8-isoprostane PGA2), 8-isoprostaglandin F2α (8-isoprostane PGF2alpha), 20-hydroxyeicosatetraenoic acid (20-HETE), and ROS concentrations at DO, CU, DIM2-10.

Data Analysis for cell culture. Prior to data analysis, variables were examined for normality distribution using histograms and Q-Q plots. If variables did not meet normality assumptions, it was transformed prior to analysis. One- or two-way ANOVA was performed using the proc mixed procedure with or without repeated measures where appropriate using the SAS software (SAS 9.4, Cary Inc., Rural Hall, NC, USA) to analyze the cell culture treatment outcomes. One-way ANOVA with Dunnett’s post hoc correction for multiple comparisons was used to analyze cell viability and mRNA expression data. A two-way ANOVA with a Tukey adjustment for multiple comparisons was used to compare the barrier integrity data for treatment and time factors and their interactions. Significance was declared when *p* ≤ 0.05.

## 3. Results

Summary statistics for the oxidative stress biomarkers and vitamins by time point are shown in Table 2.

### 3.1. Correlations between Vitamins and Oxylipids

The correlation analyses shown in Table 3 were interpreted based on ranges determined by Mukaka, 2012 [43]. Briefly, correlations between 0–0.3 have a negligible association, 0.3–0.5 have a low association, 0.5–0.7 have a moderate association, 0.7–0.9 have a high association, and greater than 0.9 have a very high association [43]. At dry off, vitamin E had a low positive correlation with ROS (r = 0.36, *p* < 0.01). Similarly, β-carotene also had a low positive correlation with serum ROS (r = 0.35, *p* < 0.05) as well as with vitamin A concentrations (r = 0.46, *p* < 0.01). β-carotene also had a high negative correlation with 8,12-isoprostane (r = −0.77, *p* < 0.05) and 8-isoprostane PGA2 (r = −0.80, *p* < 0.05). Serum vitamin A concentrations had a negligible positive correlation with serum vitamin D concentrations (r = 0.20, *p* < 0.01), a low positive correlation with serum 20-HETE (r = 0.42, *p* < 0.05), and a high positive correlation with 8-isoprostane PGA2 (r = 0.76, *p* < 0.05).

At the close-up, vitamin A concentrations had a positive negligible correlation with serum β-carotene (r = 0.19, *p* < 0.01), vitamin E (r = 0.30, *p* < 0.01), and vitamin D concentrations (r = 0.16, *p* < 0.05). Serum concentrations of β-carotene had a low positive correlation with serum vitamin E concentrations (r = 0.32, *p* < 0.01). At DIM 2-10, serum vitamin A concentrations had a negligible positive correlation with serum vitamin E concentrations (r = 0.18, *p* < 0.05) as well as with serum vitamin D concentrations (r = 0.29, *p* < 0.01). Serum vitamin A concentrations were moderately positively associated with β-carotene concentrations (r = 0.61, *p* < 0.01) and moderately negatively correlated with serum 20-HETE (r = −0.57, *p* < 0.01). β-carotene concentrations had a negligible positive correlation with serum vitamin E concentrations (r = 0.23, *p* < 0.01) and a low negative correlation with 20-HETE (r = −0.42, *p* < 0.05). When all sample time points were combined, serum concentrations of vitamins E had a low positive correlation with β-carotene concentrations (r = 0.35, *p* < 0.01), and a positive negligible correlation with vitamin A concentrations (r = 0.26, *p* < 0.01), as well as with vitamin D concentrations (r = 0.16, *p* < 0.01). Vitamin E also had a negative negligible correlation with serum 20-HETE concentrations (r = −0.23, *p* < 0.05). β-carotene concentrations had a positive low correlation with serum vitamin A concentrations and a positive but negligible correlation with vitamin D concentrations (r = 0.16, *p* < 0.01). Serum β-carotene concentration had a low negative correlation with serum 20-HETE concentrations (r = −0.42, *p* < 0.01). Serum vitamin A had a positive negligible correlation with serum vitamin D concentrations (r = 0.26, *p* < 0.01) and a negative negligible correlation with 20-HETE concentrations in the post-partum period (r = −0.25, *p* < 0.05).

### 3.2. Vitamins and AOP

There were no variables that were significantly associated with AOP at DO, therefore these results were omitted. Vitamin D concentrations were associated with AOP at CU and DIM-2-10 (Table 4). Higher vitamin D concentrations correlated to a higher concentration of AOP at both CU and DIM2-10 (*p* = 0.04 and *p* < 0.01, respectively).

### 3.3. Cell Culture

**Figure 1:**
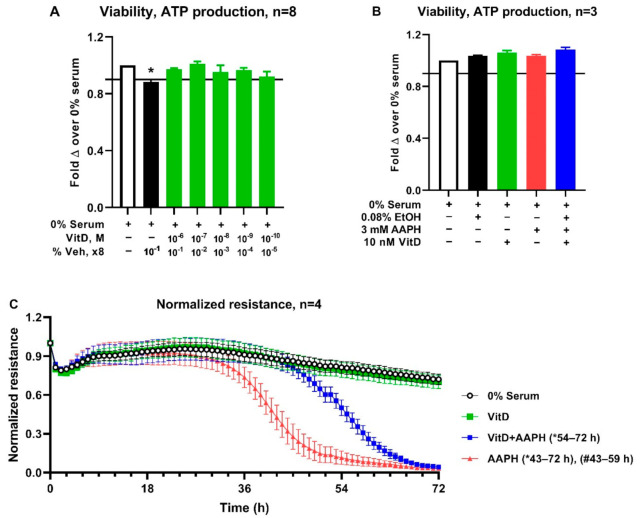
Effect of Calcitriol (vitamin D) on barrier integrity of BAEC treated with AAPH. A dose-response curve was performed to test physiological vitamin D concentrations (10^−6^ M–10^−10^ M) on cell viability for 24 h (**A**). The highest vitamin D concentration (10^−^^7^ M) maintaining at least 90% viability relative to untreated control was assessed for effect on barrier integrity in cells exposed to 3 mM AAPH (**C**). The viability of BAEC in cells exposed to treatments for the duration of barrier integrity assessment was determined (**B**). Viability data were each compared to untreated control and analyzed by the one-way ANOVA with Dunnett’s post hoc adjustment for multiple comparisons. Two-way repeated-measures ANOVA analyzed barrier integrity data with Tukey adjustment for multiple comparisons. Asterisk (*) indicates a significant difference from untreated control for viability (1A) and barrier integrity (1C). Duration of differences in barrier integrity is indicated in parentheses in C. Pound (#) shows the comparison between treatment with 3 mM AAPH with 10 nM Vit D vs. 3 mM AAPH alone. For all comparisons, α = 0.05.

**Figure 2:**
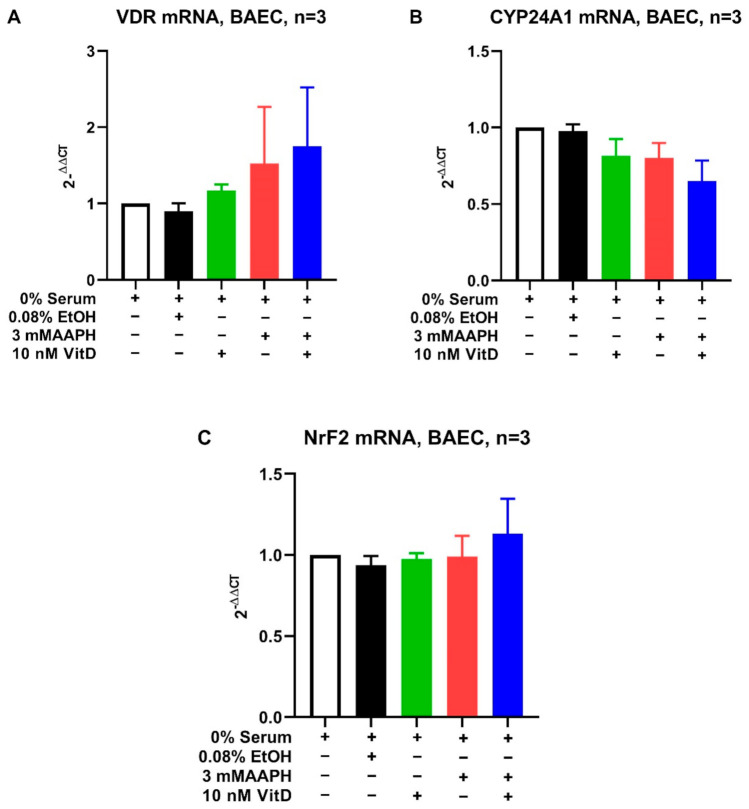
Gene expression in BAEC treated with AAPH with or without vitamin D. The mRNA expression was quantified for vitamin D receptor (VDR, (**A**)), cytochrome P 450 24A1 (CYP24A1, (**B**)), and nuclear erythroid factor 2 like (NrF2, (**C**)) after incubation with treatments for 60 h. The mRNA expression data are presented as fold change relative to untreated controls (open bars). The mRNA expression data for each treatment were compared to untreated control and analyzed by the one-way ANOVA with Dunnett’s post hoc adjustment for multiple comparisons. For all comparisons, *α* = 0.05.

Based on the positive correlation between vitamin D and AOP, the potential protective effects of vitamin D against oxidative stress were explored using an in-vitro endothelial cell model of oxidative stress. A dose titration was performed to identify a vitamin D concentration within physiologic range for dairy cattle to assess potential effects on barrier integrity of BAEC (Figure 1). Exposure of BAEC to vitamin D doses ranging from 1000 nM to 0.1 nM for 24 h maintained cell viability above 90% (Figure 1A). Treatment with vehicle alone (ethanol 0.8% by volume) which as equivalent to vehicle content in the highest vitamin D concentration tested (1000 nM) induced significant decrease (*p* < 0.05) in cell viability relative to untreated control (Figure 1A). Consequently, the highest dose of 10 nM of vitamin D (which contained 0.08% ethanol) was selected for assessing effects of vitamin D on barrier integrity in BAEC exposed to 3 mM AAPH. The 10 nM of vitamin D maintained BAEC viability at levels similar to untreated controls for the duration closer to that of the barrier integrity assessment (Figure 1B). The presence of VDR in BAEC was confirmed, however, mRNA expression for VDR was not affected by treatment (Figure 2). Similarly, neither CYP24A1 nor NrF2 differed among treatments (Figure 2). The treatment of BAEC with vitamin D alone did not alter barrier integrity relative to untreated controls. Barrier integrity was decreased by treatment with AAPH. Pretreatment of BAEC with vitamin D significantly (*p* < 0.05) prevented the loss of barrier resistance induced by AAPH by 16 hours (Figure 1C).

## 4. Discussion

Dietary supplementation of vitamins and minerals are commonly provided to cattle at concentrations greater than required for normal physiological processes to enhance immune function and decrease the risk for oxidative stress [7]. Indeed, vitamin E, β-carotene, and selenium are expressly formulated into rations to promote antioxidant defenses, particularly in periparturient period [16]. Surprisingly, the current study found that only vitamin D, but not vitamin E or β-carotene, was positively associated with AOP at both the CU and DIM2-10. These results were surprising because vitamin E and β-carotene have antioxidant function [44]. Vitamin D, conversely, is well known for its role in promotion of gene transcription and regulation of calcium, however current literature on the effects of vitamin D on oxidative stress in cattle is still limited [45,46]. These results indicate that vitamin D may have a role in regulating the oxidant status in cows and it should be more fully investigated.

The increased risk for oxidative stress in periparturient period is a widespread challenge in the dairy industry negatively affecting milk production and cow welfare through increased risk of disease [47]. Vitamins E and β-carotene with antioxidant functions have decreased plasma concentrations during the periparturient period due to vitamin secretion in colostrum, decreased intake of vitamin-supplemented feed, and increased degradation from ROS [24,26]. For this reason, increased dietary supplementation of antioxidants, such as vitamin E, is employed to decrease risk of oxidative stress in the periparturient period [8]. Merely increasing antioxidant supplementation may not be effective. For example, Bouwstra et al. demonstrated that dietary supplementation of vitamin E at 3 times recommended concentrations increased oxidative stress and mastitis incidence in dairy cows [48]. This paradox is demonstrated at the dry-off time point where both vitamins E and β-carotene concentrations are positively correlated with serum ROS (r = 0.35, 0.35, *p* < 0.5 respectively). Indeed, vitamin E had no other significant association with any other biomarker of oxidative stress at the individual time points in this study, including the periparturient period (*p* > 0.05).

Vitamins A and D function more like hormones, affecting transcription of a wide variety of genes. Therefore, vitamins A and D are not antioxidants by definition, but both demonstrate antioxidant effects in cattle in response to supplementation [21,49,50]. For example, vitamin D treatment can alter antioxidant potential indirectly by increasing glutathione peroxidase concentrations in humans [46]. This is important because total AOP is composed of 3 elements: enzymes such as glutathione peroxidase, non-enzymatic protein antioxidants primarily with sulfhydryl groups, and low molecular weight molecules such as glutathione, vitamin E, and β-carotene [51]. The association between serum vitamin D concentrations and AOP at CU and DIM 2-10 may be a result of increases in AOP components from vitamin D gene transcription [52].

The Spearman correlation analysis reveals interesting insights into the relationships of serum vitamins and biomarkers of oxidative stress and the most significant finding is the negative correlation between 20-HETE concentrations with serum vitamin A and β-carotene concentrations at DIM2-10 and in the combined time point analysis. The oxylipid 20-HETE is derived from cytochrome P-450 metabolism of arachidonic acid [53]. 20-HETE is not only increased in serum during the periparturient period but is also elevated in serum and milk during severe coliform mastitis [32,54]. Indeed, 20-HETE induces oxidative stress, while vitamin A and β-carotene improve antioxidant status [21,55,56]. Elevated serum 20-HETE concentrations during the periparturient period concentrations may occur in part due to a depletion of vitamin A and β-carotene serum concentrations and thus a decrease in free radical quenching capacity. Serum vitamin E concentrations were negatively correlated with serum 20-HETE concentrations when all time points were combined as well. Kuhn et al. found that vitamin E could reduce 20-HETE production through competitive cytochrome metabolism with arachidonic acid [57]. These findings indicate that redox balance is more complicated than quenching of free radicals and the relationships between serum vitamins and 20-HETE concentrations merits further investigation.

The association between serum concentrations of vitamin D at CU and DIM2-10 is particularly interesting because vitamin D is not a direct antioxidant and thus the mechanism behind the association is not immediately evident. The functional significance of the relationship between vitamin D and AOP was investigated using an in-vitro model of oxidative stress using bovine aortic endothelial cells. Vascular endothelial cells are essential in regulating inflammatory responses and orchestrating the barrier integrity between the circulating blood and local tissues [58]. Damage to endothelial cells through oxidative stress may lead to aberrant inflammation and a dysfunctional endothelial cell barrier [59]. The model of oxidative stress using a free radical generator, AAPH, was previously reported by our group as inducing loss of barrier integrity in BAECs [39]. Thus, the effect of vitamin D on the loss of barrier integrity in this model was assessed. We demonstrated that vitamin D could prevent the functional disruption of endothelial cell barrier resistance with an in-vitro model of prooxidant challenge. Our findings agree with previous studies that showed that vitamin D improved barrier integrity in endothelial progenitor cells during tumor necrosis factor α-induced inflammatory conditions [59]. Although the mechanism was not investigated in our studies, inhibition of pro-oxidant generation pathways may be responsible for the protective effects on barrier integrity [60,61]. It is also unclear if the protective effects on barrier integrity of the BAECs are meditated via the vitamin D receptor (VDR) because of a lack of differences in mRNA expression on exposure to vitamin D. However, other studies reported presence of VDR in BAECs and that mRNA expression increases upon exposure to vitamin D [52].

The current study also did not find mRNA expression changes of CYP24A1, the enzyme responsible that metabolizes vitamin D. The CYP24A1 gene expression generally increases upon exposure to vitamin D in BAECs or other cells [62]. These findings suggest that vitamin D may protect endothelial barrier integrity through a mechanism independent of transcriptional regulation. The fact that vitamin D was protective in a prooxidant challenge suggests a direct or indirect effect on antioxidant protective mechanisms against oxidative stress and changes in redox balance. Other oxidative stress models in cell culture systems report augmentation of antioxidant response systems, increased reducing capacity and decreased formation of isoprostanes following treatment with vitamin D [63]. Follow-up studies will investigate the specific nature of the correlation between vitamin D and AOP and the mechanisms responsible for maintaining barrier integrity of BAEC in vitro.

Our in-vivo study showed a significant positive correlation between serum vitamin D concentrations and serum AOP in periparturient cattle at high risk for oxidative stress. Dairy cattle are at an increased risk of developing inflammatory disorders after calving [19]. Our in-vitro prooxidant challenge did not evaluate changes in antioxidant potential but assessed changes in the gene expression for the master regulator of antioxidant responses, NrF2 [52]. The lack of effects in the expression of NrF2 likely further supports that AAPH treatments did not induce further prooxidant challenge commonly affecting cell culture systems [64]. Since the assessment of Nrf2 was assessed at 60 h, this precluded the identification of potential early changes in gene expression of NrF2. Future studies should assess antioxidant potential, temporal changes in NrF2 and other specific antioxidant response genes to understand the mechanism by which vitamin D exerts its endothelial stabilizing effects.

## 5. Conclusions

Serum concentrations of vitamin D are positively associated with AOP in periparturient cattle, but the common antioxidant vitamins including vitamins A, E, and β-carotene are not associated with AOP in the current study. The results of this study indicate that the relationship between vitamins A, D, E, and β-carotene and oxidative stress must be re-evaluated. As the understanding of oxidative stress in dairy cattle continues to grow, re-examination of the effectiveness of common oxidative stress-mitigation strategies such as dietary vitamin supplementation is paramount. The re-examination of oxidative stress-mitigation strategies is particularly salient because despite increased supplementation of vitamins such as vitamin E in the periparturient period, oxidative stress and periparturient diseases remain a consistent problem in the dairy industry. The potential benefits of supplementary vitamin D on serum AOP could have a major impact on prevention of oxidative stress and disease periparturient cows and therefore further research is needed.

## Figures and Tables

**Table 1:** Proprietary TaqMan primer reference information.

Gene	NCBI Reference Sequence ^1^	TaqMan Assay ID
CYP24A1	NM_001191417.1	Bt04306544_m1
NFE2L2	NM_001011678.2	Bt03251878_m1
VDR	NM_001167932.2	Bt04301663_m1
ACTB	NM_173979.3	Bt03279174_g1
GUSB	NM_001083436.1	Bt03256165_m1
RPS9	NM_001101152.2	Bt03272017_m1

^1^ National Center for Biotechnology Information reference sequence found in the nucleotide da-tabase (https://www.ncbi.nlm.nih.gov/nuccore/, accessed 4 January 2021). CYP24A1, Cytochrome P24A1; NFE2L2, Nuclear erythroid factor 2 like 2; VDR, Vitamin D receptor; ACTB, β-actin; GUSB, Glucuronidase beta; RPS9, Ribosomal protein S9.

**Table 2:** Summary statistics of oxidative stress biomarkers and vitamins by time point (N = 240 cows).

Biomarker	Time Point	N	Mean	SEM	Min	25th Percentile	Median	75th Percentile	Max
Vitamin E (μg/mL)	DO	239	2.66	0.06	0.71	1.99	2.60	3.29	5.28
	CU	214	3.12	0.10	0.61	2.02	2.78	4.06	9.44
	DIM 2-10	198	1.79	0.06	0.001	1.15	1.81	2.37	4.57
AOP (TE/μL)	DO	240	5.08	0.13	1.31	3.57	4.69	6.43	10.80
	CU	222	5.05	0.13	2.38	3.59	4.57	5.83	10.80
	DIM 2-10	207	4.36	0.10	1.05	3.38	4.27	5.20	8.63
β-carotene (μg/mL)	DO	238	5.00	0.25	0.21	2.62	4.07	6.00	23.62
	CU	221	3.35	0.16	0.10	1.77	2.62	4.53	13.87
	DIM 2-10	206	1.63	0.09	0.10	0.74	1.15	2.02	6.39
ROS (CarrU/μL)	DO	35	169.40	10.44	16.3	130.7	155.2	212.3	310.3
	CU	12	132.71	11.43	49.0	110.3	138.8	155.2	204.2
	DIM 2-10	22	181.52	8.80	122.5	155.2	175.6	212.3	277.7
Vitamin A (ng/mL)	DO	239	302.63	5.41	102	238	296	358	528
	CU	221	277.15	5.88	112	213	278	331	561
	DIM 2-10	205	256.81	8.33	53	177	241	316	764
Vitamin D (ng/mL)	DO	186	99.12	2.02	25.40	81.0	97.60	112.50	198.60
	CU	173	93.77	2.19	20.10	77.20	94.80	110.00	210.40
	DIM 2-10	184	82.06	1.71	9.80	63.95	82.30	96.30	166.40
5- iPF2alphaVI (ng/L)	DO	7	0.07	0.01	0.05	0.05	0.05	0.10	0.10
	CU	7	0.06	0.01	0.05	0.05	0.05	0.05	0.10
	DIM 2-10	7	0.06	0.01	0..05	0.05	0.05	0.10	0.10
8,12-isoprostane (ng/L)	DO	7	0.26	0.05	0.1	0.10	0.30	0.40	0.40
	CU	7	0.23	0.07	0	0.10	0.20	0.30	0.60
	DIM 2-10	7	0.21	0.04	0.1	0.10	0.20	0.30	0.40
8-isoprostane PGA2 (ng/L)	DO	7	0.29	0.05	0.10	0.20	0.30	0.40	0.50
	CU	7	0.73	0.13	0.40	0.50	0.60	0.90	1.4
	DIM 2-10	7	0.16	0.04	0.10	0.10	0.10	0.20	0.40
8-isoprostane PGF2alpha (ng/L)	DO	7	0.26	0.15	0	0	0.10	0.30	1.1
	CU	7	0	0	0	0	0	0	0
	DIM 2-10	7	0.73	0.17	0	0.50	0.70	1.0	1.5
20-HETE (μg/L)	DO	27	2.22	0.27	0.01	1.20	1.90	3.19	6.54
	CU	24	5.28	1.66	0.28	1.71	3.60	5.75	41.41
	DIM 2-10	27	9.31	2.31	0.50	2.78	4.80	8.50	51.95

AOP = antioxidant potential, ROS = reactive oxygen species, CarrU = Carratelli units, 5-isoprostaglandin F2α VI (5-iPF2alphaVI), 8,12-isoprostane, 8-isoprostane prostaglandin A2 (8-isoprostane PGA2), 8-isoprostaglandin F2α (8-isoprostane PGF2alpha), 20-hydroxyeicosatetraenoic acid (20-HETE)DO = dry off, CU = close up, DIM2-10 = 2–10 days in milk.

**Table 3:** Correlation analysis results for vitamins, oxylipids. 20-HETE, and ROS.

Dry Off										
	Vitamin E	β-carotene	ROS	Vitamin A	Vitamin D	5-iPF2alphaVI	8,12-isoprostane	8-isoprostane PGA2	8-isoprostane PGF2alpha	20-HETE
Vitamin E	1									
β-carotene	0.04	1								
ROS	0.36 **	0.35 *	1							
Vitamin A	0.08	0.46 **	0.3	1						
Vitamin D	0.08	−0.002		0.20 **	1					
5-iPF2alphaVI	0.43	−0.43	0.80 *	0.43		1				
8,12-isoprostane	−0.13	−0.77 *	0	0.4		0.07	1			
8-isoprostane PGA2	−0.13	−0.80 *	−0.19	0.76 *		0.15	0.74	1		
8-isoprostane PGF2alpha	−0.07	0.15	0.2	−0.3		0.15	−0.56	−0.28	1	
20-HETE	−0.32	0.12	0.2	0.42 *	−0.04	0.29	−0.78 *	−0.35	0.27	1
Close up										
Vitamin E	1									
β-carotene	0.32 **	1								
ROS	0.16	−0.03	1							
Vitamin A	0.30 **	0.19 **	0.07	1						
Vitamin D	0.19 *	−0.04		0.16 *	1					
5-iPF2alphaVI	0.39	−0.61	0.77	0		1				
8,12-isoprostane	0.35	−0.11	0.2	−0.38		0.62	1			
8-isoprostane PGA2	−0.17	−0.67	0.95	−0.04		0.62	0.33	1		
8-isoprostane PGF2alpha								0.37 *	1	
20-HETE	−0.38	0.15	0.4	−0.12	0.19	−0.41	−0.33	0.14		1
DIM2-10										
Vitamin E	1									
β-carotene	0.23 **	1								
ROS	0.16	−0.13	1							
Vitamin A	0.18 *	0.61 **	0.01	1						
Vitamin D	0	0.07		0.29 **	1					
5-iPF2alphaVI	−0.39	−0.32	0.48	0.39		1				
8,12-isoprostane	−0.03	−0.62	0.05	0.76		0.08	1			
8-isoprostane PGA2	0.39	0.18	0.23	0.65		0.39	−0.19	1		
8-isoprostane PGF2alpha	−0.26	−0.54	0.73	0.66		0.79 *	0.24	0.37 *	1	
20-HETE	0.04	−0.42 *	−0.02	−0.57 **	−0.4	0.79 *	−0.13	0.04	0.46	1
All time points										
Vitamin E	1									
β-carotene	0.35 **	1								
ROS	0.03	0.14	1							
Vitamin A	0.26 **	0.45 **	0.24	1						
Vitamin D	0.16 **	0.16 **		0.26 **	1					
5-iPF2alphaVI	0.26	−0.31	0.65 **	0.25		1				
8,12-isoprostane	0.05	−0.3	0.08	0.23		0.3	1			
8-isoprostane PGA2	0.42	0.09	−0.23	−0.05		0.05	0.13	1		
8-isoprostane PGF2alpha	−0.45	−0.38	0.42	0.21		0.24	−0.1	0.37 *	1	
20-HETE	−0.23 *	−0.42 **	0.13	−0.25 *	−0.19	−0.02	−0.4	−0.07	0.23	1

* *p* < 0.05, ** *p* < 0.01, Missing values: not enough data points to calculate correlation coefficient, 5-isoprostaglandin F2α VI (5-iPF2alphaVI), 8,12-isoprostane, 8-isoprostane prostaglandin A2 (8-isoprostane PGA2), 8-isoprostaglandin F2α (8-isoprostane PGF2alpha), 20-hydroxyeicosatetraenoic acid (20-HETE), reactive oxygen species (ROS).

**Table 4:** Mixed linear regression results for the association of vitamin D with AOP at CU and DIM2-10.

Variable	Coefficient	L95% CI	U95% CI	SE	z	*p*-Value
CU ^1^ (*N* = 173)						
Vitamin D	0.006	0.0001	0.01	0.003	2.01	0.04
Intercept	3.79	3.13	4.46	0.34	11.18	<0.01
DIM2-10 ^2^ (*N* = 184)						
Vitamin D	0.008	0.003	0.01	0.003	2.96	0.01
Intercept	3.43	2.82	4.03	0.31	11.15	<0.01

^1^ Random intercept estimate for Cohort: 0.84, 95% CI (0.59, 1.19). ^2^ Random intercept estimate for Cohort: 0.83, 95% CI (0.57, 1.20). CU = close up, DIM2-10 = 2–10 days in milk, AOP = antioxidant potential.

## Data Availability

All data generated or analyzed during this study are included in this published article.
